# Supplementary material for: A Global Screen for Assembly State Changes of the Mitotic Proteome by SEC-SWATH-MS
Source: Cell Syst. 2020 Feb 26;10(2):133–155.e6. doi: 10.1016/j.cels.2020.01.001 (PMC7042714; doi:10.1016/j.cels.2020.01.001)

Q6FIF0 | ZFAN6_HUMAN | ZFAND6 AWP1 ZA20D3 HT032
Monomer MW [kDa]: 22.555 Monomer expected elution fraction: 53

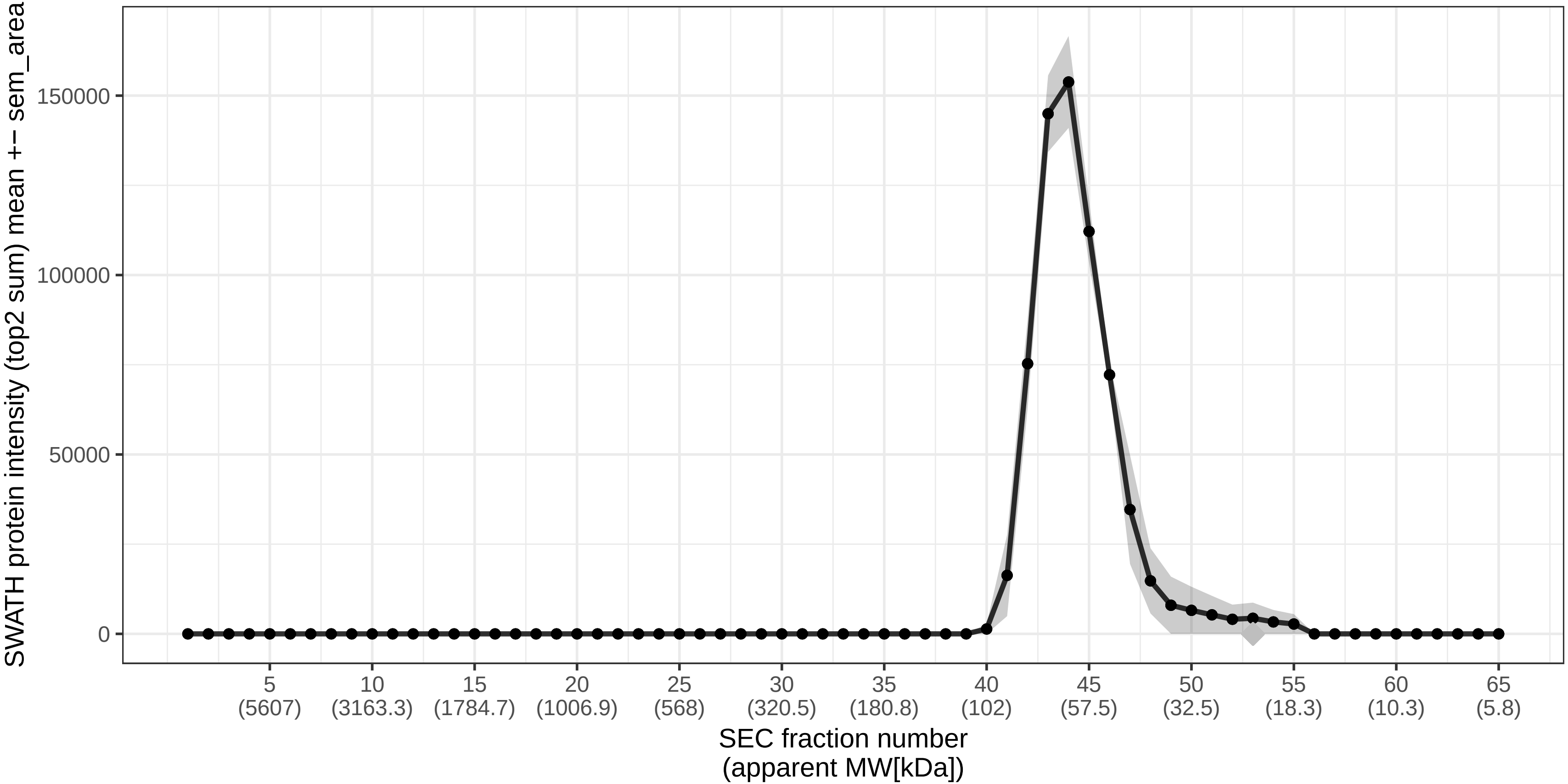

Supplement: Data S1. SEC-SWATH-MS Protein Chromatograms, Related to Figure 1 [file mmc6.zip › SECchrom_Q6FIF0_ZFAN6_HUMAN_ZFAND6_AWP1_ZA20D3_HT032.pdf]
